# Can an Eight-Session Multicomponent Physical Exercise Program Reduce Fall Risk and Fear of Falling among the Elderly?

**DOI:** 10.3390/ijerph19148262

**Published:** 2022-07-06

**Authors:** Antony G. Philippe, Aurélie Goncalves, Christophe Martinez, Maxime Deshayes, Elodie Charbonnier

**Affiliations:** UNIV. NIMES, APSY-V, F-30021 Nîmes Cedex 1, France; aurelie.goncalves@unimes.fr (A.G.); christophe.martinez1@unimes.fr (C.M.); maxime.deshayes@unimes.fr (M.D.); elodie.charbonnier@unimes.fr (E.C.)

**Keywords:** fall risk, fear of falling, aging population, balance, physical exercise

## Abstract

In older populations, falls are responsible for decrease autonomy and increased pain and injuries. With aging, fall risk is multifactorial and associated with sarcopenia, impaired balance, falls themselves and psychological factors such as fear of falling. The objectives of the present study were: (a) to test the effects of a short multicomponent physical exercise program on fall risk and fear of falling; and (b) to analyze the relationship between fall risk and fear of falling. The participants were thirty-five older persons who were engaged in an eight-session physical exercise program. Balance (i.e., One-leg Balance Test, and Functional Reach Test), lower-limb endurance (i.e., Wall-sit Test) and fear of falling were measured before and after the multicomponent physical exercise program. Results indicated an increase in balance and a diminution of fear of falling after the end of the physical exercise program (*p* < 0.05). The program has an effect on lower limb endurance (*p* > 0.05). Gains in balance were correlated to the diminution of fear of falling (*p* < 0.05). An 8-week multicomponent physical exercise program based on balance is efficient to reduce fall risk and fear of falling among the elderly.

## 1. Introduction

Among the elderly, fall risk is associated with a reduction in quality of life and mobility [1,2]. Indeed, falls are an important cause of fatal and nonfatal injuries among older adults [3,4]. Approximately 28.7% of older adults reported a least one fall in the preceding 12 months, resulting in an estimated 29.0 million falls and 7.0 million fall injuries in the United States [5]. Moreover, fall risk and related injuries increase with age [6,7,8], which can be explained by different factors: the degradation of skeletal muscle system, i.e., sarcopenia [9,10,11] and quadriceps muscle strength [12], sensorial factors (e.g., vision [13]) environment (e.g., home and environmental hazards [14]) and fear of falling [15]. Global self-esteem and fall history are associated with a higher fear of falling in elderly [16]. Therefore, the establishment of prevention programs appears to be essential in order to reduce falls risk among the elderly.

To date, several strategies have been proposed to prevent fall risk and functional decline of the body with aging. These strategies include muscular reinforcement with resistance exercises, balance exercises, blood flow restriction and neuromuscular electrical stimulation or vibrations [12,17,18,19,20]. Numerous studies have shown that exercise training interventions ameliorate muscle function and reduced the risk of falls [21]. Nevertheless, these interventions typically last several months since observed gains in balance are associated to physical adaptations. For example, a 16-week resistance training program based on descending a four-step stair could confer functional improvements in older adults when descending a staircase [22]. A recent study by Liu-Ambrose and collaborators has shown a reduction of incidence rates of falls per person-year in older people after 12 months of home-based strength and balance retraining exercise program [23]. Ansai and collaborators compared the effects of 16-week multicomponent and resistance training on physical variables related to a higher risk of falls in very old people [24]. The results indicated that after a 16-week intervention period, the multicomponent group had a significant improvement in balance. Skeletal muscle reinforcement base on multicomponent training is considered as a protective factor for fall risk. Overall, physical activity programs have shown an amelioration of muscle function among the elderly.

However, physical condition is not the only factor that influences fall risk. Psychological factors and especially fear of falling seems to be an important predictor of falls among the elderly. Indeed, a study by Lavedán and collaborators showed that fear of falling is a cause and a consequence of falls [15]. Authors reported that 41.7% of the subjects who had reported a fear of falling at baseline had suffered at least one fall 24 months later. Beyond effects on physical conditions, intervention programs among the elderly have effects on psychological factors such as fear of falling. It has been shown that fear of falling was reduced and associated to an increase in dynamic balance after a 12-weeks balance-based program with two sessions a week among the elderly [25].

Thus, beyond muscular reinforcement and balance, the evaluation of fear of falling seems to be an important measure in fall risk prevention among the elderly [26]. Indeed, experiences of falling during the previous month or the previous year are both associated with an important fear of falling [27]. Nevertheless, most of the studies focused on falls risk and fear of falling are based on long physical activity intervention programs that allow skeletal and skeletal muscles adaptations [28,29]. The study of Leiros-Rodríguez and Garcia-Soídan indicates that a short 12-sessions program with two sessions a week can improve balance [30]. Data are missing concerning the effects of short physical activity intervention programs on fall risk and fear of falling. Thus, the aim of the present study was to test whether an eight-session multicomponent physical exercise program with one session-a-week based on balance reinforcement can reduce falls risk and fear of falling in the elderly. We hypothesized that a multicomponent physical exercise program will decrease (1) fall risk and (2) fear of falling and that (3) decreased fall risk will be associated with decreased of fear of falling.

## 2. Materials and Methods

### 2.1. Subjects

Of the seventy-three initial subjects, only thirty-five of them completed. Subjects were 66 to 89 years of age (76.3 ± 7.9 years old) and were engaged in the program. Dropouts were due to medical (disease, injury) and personal reasons (family). Subject were recruited in the Nîmes Sport-Santé association, France, that proposes adapted physical activities and sensitization programs. To participate, subjects had to be member of the association, have a good autonomy, living at home, aged >65 years old and signed informed consent. The exclusion criteria were residential care and terminal illness. Authors of the present study had the written authorization of both participants and Nîmes Sport-Santé association to use the data collected by the association.

### 2.2. Multicomponent Physical Exercise Program

The program was composed by 8, one-hour weekly morning sessions supervised by an adapted physical activity teacher. Subjects were divided into two groups of 5–15 people. The content was specific to each session. In sessions 1 and 3, subjects realized balance exercises. Sessions 2 and 5 consisted of upper and lower limb muscle strengthening exercises. Sessions 4 and 6 focused on gesture and posture. During session 7, the subjects worked on proprioception. Session 8 was reserved for a general review of the exercises covered in previous sessions and to give advice and tools for independent practice. The program is fully detailed in Table 1.

### 2.3. Balance and Fall Risk Assessments

Pre and post balance and fall risk assessments were completed before and after the intervention program by using a combination of balance and physical tests.

#### 2.3.1. One-Leg Balance Test

Equilibration level was measured with the one-leg balance test, which has been shown to be an important predictor of falls among older adults [31]. It consists of holding a standing position on one leg (left, then right) with eyes open and closed. Subjects were asked to hold the position as long as possible and time was recorded. Holding the position for less than 5 sec with eyes open is considered to be an important indicator of risk of falling [31].

#### 2.3.2. Wall-Sit Test

The wall-sit test is a test of lower body muscular strength and endurance. The test requires the subject to hold a sitting position while leaning back against a wall with their knee bent at 90° and time was recorded.

#### 2.3.3. Functional Reach Test

Functional reach test (FRT) is a clinical measure, defined as the maximum distance one can reach, forward beyond arm’s length. It is used to evaluate elderly subjects at risk of recurrent falls and measure dynamic balance ability clinically. Forward distance was typically between 16 and 35 cm [32].

### 2.4. Evaluation of Fear of Falling

Fear of falling was evaluated by participants answering a question “are you afraid of falling?” on a one-to-ten Likert-scale. One being the lowest fear of falling and ten the maximum. Participants reported their fear of falling on the analogic scale twice, during the first and the last session of the intervention program. Thus, 22 subjects were retained for the evaluation of fear of falling.

### 2.5. Statistical Analysis

Variable data normality was checked using the Shapiro–Wilk test and homogeneity of variance with the F-test. Given that the studied variables displayed a deviation from normality, effects were analyzed by non-parametric tests. Thus, the paired-sample Wilcoxon test was used to compare pre- and post-evaluations. Pearson correlation was performed to test the association between the reduction of fear of falling and the amelioration of static balance before and after the intervention program. Analyses were performed using GraphPad Prism software (Prism v.6; GraphPad Software, La Jolla, CA, USA). Data are presented as means ± SD. Significance was set at *p* < 0.05.

## 3. Results

### 3.1. Effects of the Multicomponent Physical Exercise Program on Fall Risk

#### 3.1.1. Endurance

Endurance in lower limb was evaluated by the wall-sit test. Endurance was not improved after the program. Indeed, the time subjects could hold in the wall-sit position was 60.7 ± 4.1 sec and 59.1 ± 5.0 sec before, and at the end of the program, respectively (*p* > 0.05) (Table 2).

#### 3.1.2. Balance

Subjects performed the functional reach test during the first and the last session. Analysis of results showed a significant increase in the forward distance subjects could reach beyond arm’s length. Table 2 presents a significant gain of 13.1% of the maximal forward distance reached, i.e., 26.1 ± 1.1 cm vs. 29.5 ± 0.9 cm before and after the program, respectively (*p* < 0.001).

One-leg balance test was improved by the program whatever the condition, i.e., with eyes open and closed. Indeed, in the condition with eyes open, the maximal time subjects could hold in a standing position increased by 39.2% with the right foot on the floor, i.e., 16.2 ± 2.0 s vs. 22.3 ± 1.8 s before and after the program, respectively (*p* = 0.01) and increased by 24.8% with left foot on the floor, i.e., 13.9. ± 2.0 s vs. 17.3 ± 2.0 s before and after the program, respectively (*p* = 0.026). In the condition with eyes closed, the maximal time subjects could hold in a standing position increased by 98.6 % with the right foot on the floor, i.e., 3.2 ± 0.2 s vs. 6.3 ± 1.2 s before and after the program, respectively, (*p* < 0.001) and increased by 179.6% with the left foot on the floor, i.e., 2.4 ± 0.4 s vs. 6.7 ± 1.1 s before and after the program, respectively (*p* < 0.001).

### 3.2. Effects of the Multicomponent Physical Exercise Program on Fear of Falling

Table 2 indicates fear of falling before and after the program. Results indicate that fear of falls was significantly reduced. Indeed, it decreased from 4.9 ± 0.6 to 3.7 ± 0.5 before and after the program, respectively (*p* = 0.036).

### 3.3. Balance Is Associated to Fear of Falling

A correlation has been performed to check the association between the reduction of fear of falling and balance indicators, i.e., amelioration of functional reach test and one-leg balance test (eyes open and closed) between the beginning and the end of the program. To this end, gains in balance indicators and reduction of fear of falling have been calculated from the difference of values between the end and the beginning of the program. Results are presented in Table 3. It appears that the gain in the functional reach test and in the time that the participant could hold on the left foot with eyes open are significantly correlated to the reduction of fear of falling (*p* = 0.026 and *p* = 0.039, respectively). Other balance indicators are not significantly correlated to the reduction of fear of falling (*p* > 0.05).

## 4. Discussion

In this study, we test whether an eight-session multicomponent physical exercise program based on balance and muscular reinforcement could reduce fall risk and fear of falling among the elderly. Several results generated from this study are worthy of discussion.

With aging, more and more people are likely to fall. Prevalence of fear of falling is between 20–39% among community-dwelling older adults [33,34,35]. These falls have important physiological and psychological consequences [36,37,38]. After an experience of fall, the frailty of elderly increases [39], reducing autonomy, physical and social activities and quality of life [40]. In addition, an augmentation of fear of falling is associated with an increase in risk of fall, while low levels of fear of falling appear to have protective effects on risk of fall, regardless of the presence of balance disorders [41]. Our results are in line with the literature and suggest that amelioration of balance after the physical activity program is associated to the reduction of fear of falling [15,42]. According to the correlation matrix, this suggests that under our conditions, the reduction of falls risk (i.e., improvement of balance in the functional reach test and one-leg balance test) is directly associated to the decrease in fear of falling.

Many studies focusing on the prevention of falls among the elderly have examined effects of physical activity programs on balance. Indeed, it has been shown that a balance-based physical activity program was associated to a reduction of fall risk [25,43,44]. Quadriceps strength has been shown to be directly related to the fall risk among the elderly, suggesting that strengthening the quadriceps muscle could reduce fall risk [45]. Thus, resistance training (RT) appears to be an efficient strategy to improve muscle function and prevent sarcopenia among the elderly [17]. RT induces the activation of anabolic cellular pathways (such as mammalian target of rapamycin mTOR) by increasing both mechanical and metabolic stress [46,47] inducing hypertrophy through increased muscle protein synthesis [48]. However, most of these studies show beneficial effects after long physical activity programs. According to Cadore and Izquierdo, these RT programs should contain at least 3 sessions a week [49]. In general, improvements in strength and muscle power in the elderly are observed after training programs lasting between 6 and 24 weeks, with at least 2 sessions a week [50,51,52,53,54]. In our study, the program was a 1 h-session a week for 8 weeks. Duration and intensity of the program seem insufficient to induce muscle adaptations that could explain the gains observed. This is in accordance with our results, since we did not observe any improvement in the wall-sit test between before and after the intervention (*p* > 0.05). Here, balance gains could not be explained by skeletal muscle adaptation. These data underline the interaction between balance and fear of falling observed our study and confirmed by Pearson’s correlation. Thus, in our conditions, balance gains could be explained by psychological aspects, i.e., reduction of fear of falling, suggesting that fear of falling is a predictor of balance among the elderly.

Subjects were asked to be more active in daily life. However, physical activity performed between sessions was not evaluated. An increased physical activity level between sessions could have explained, at least in part, the observed gain in balance in the present study.

Balance gains and decreased fear of falling should be interpreted carefully. We do not have any information on long-term effects. In order to strengthen our hypotheses, it would have been interesting to have a measure of fear of falling and balance several weeks after the end of the physical exercise program. It is possible that psychological benefits decrease over time if no follow-up is done. Under these conditions, several investigations are needed to find out the long-term effects of an eight-session multicomponent physical exercise program on balance and fear of fall among the elderly. Moreover, the use of the FES-I questionnaire to evaluate fear of falling would have been interesting as it use among the elderly and includes environmental factors [42]. Nevertheless, interaction between fear of falling and balance needs a close attention. In the construction of multicomponent physical exercise programs based on fall prevention among the elderly, it seems important to always take into account fear of falling, regardless of the training load foreseen in the program. Beyond physical activity, providing psychological support and reassurance to subjects can yet determine some benefits in balance, and thus, reduce fall risk and improve quality of life among the elderly.

## 5. Conclusions

This study showed that an 8-week multicomponent physical exercise program could increase balance and decrease fear of falling but was insufficient to improve muscle strength. Increased balance was therefore associated with decreased fear of fall rather than with muscle strengthening. Thus, psychological factors may have benefits on balance. Longer interventions should be proposed to include muscle function improvement. A global approach including both physical, psychological and environmental factors is recommended to prevent fall risk among the elderly.

## Figures and Tables

**Table 1:** Description of the multicomponent physical exercise program.

Session Number	Type of Exercises	Content
Sessions 1 and 3	Balance exercises	Balance course with strides, hoops, step on foam slab
Sessions 2 and 5	Upper and lower limb muscle strengthening exercises	Lower limb: Lunges, squats, Upper limb: Exercises with rubber band
Sessions 4 and 6	Gesture and posture exercises	Yoga posture (tree pose, standing forward bend twist pose, exalted crescent lunge pose)
Session 7	Proprioceptive training	One-leg stance with eyes open and closed, swiss ball exercises
Session 8	General review of exercises covered in previous sessions	Balance course, swiss ball exercises, lower limb muscle strengthening exercises, one-leg stance

**Table 2:** Effects of the program on endurance, static balance and risk of falls.

	Mean (SD)		*p*
	Pre-Program	Post-Program	
**Endurance (Wall-sit test)**	60.7 (24.5)	59.1 (29.4)	0.83
**Static balance**			
Functional reach test	26.1 (6.2)	29.5 (4.9)	<0.001
One-leg balance test			
RFO	16.0 (11.6)	22.3 (10.2)	0.01
RFC	3.2 (3.2)	6.3 (6.7)	<0.001
LFO	13.9 (11.4)	17.3 (11.5)	0.026
LFC	2.4 (2.6)	6.7 (6.5)	<0.001
**Fear of falling**	4.9 (2.8)	3.7 (2.5)	0.036

Abbreviations: RFO, right foot with eyes open; RFC, right foot with eyes closed; LFO, left foot with eyes open; LFC, left foot with eyes closed.

**Table 3:** Correlations between balance indicators and fear of falls.

Variables	1	2	3	4	5	6
1. delta RFO						
2. delta RFC	0.003					
3. delta LFO	0.246	−0.122				
4. delta LFC	0.198	0.113	0.04			
5. delta FRT	−0.289	−0.302	−0.243	−0.210		
6. delta FOF	−0.207	−0.231	0.443 *	0.131	0.474 *	

Abbreviations: RFO, right foot with eyes open; RFC, right foot with eyes closed; LFO, left foot with eyes open; LFC, left foot with eyes closed; FRT, functional reach test; FOF, Fear of falling. * *p* < 0.05.

## Data Availability

The data used in this research are available on request from the corresponding author. The data are not publicly available due to restrictions.
